# Crigler-Najjar syndrome type II in a Chinese boy resulting from three mutations in the bilirubin uridine 5′-diphosphate-glucuronosyltransferase (*UGT1A1*) gene and a family genetic analysis

**DOI:** 10.1186/1471-2431-14-267

**Published:** 2014-10-15

**Authors:** Bixia Zheng, Guorui Hu, Jin Yu, Zhifeng Liu

**Affiliations:** Department of Gastroenterology, Nanjing Children’s Hospital Affiliated to Nanjing Medical University, Nanjing, 210008 China; Department of Pediatric Endocrinology and Genetic Metabolism, Xinhua Hospital, Shanghai Institute for Pediatric Research, Shanghai Jiao Tong University School of Medicine, Shanghai, 200092 China

**Keywords:** Crigler-Najjar syndrome type II, *UGT1A1*, Genetic analysis

## Abstract

**Background:**

The *UGT1A1* gene encodes a responsible enzyme, UDP-glucuronosyltransferase1A1 (*UGT1A1*), for bilirubin metabolism. Many mutations have already been identified in patients with inherited disorders with unconjugated hyperbilirubinemia, such as Crigler-Najjar syndromes and Gilbert’s syndrome.

**Case presentation:**

In this report, we presented a boy with intermittent unconjugated hyperbilirubinemia, whose genetic analysis showed a new compound heterozygote determined by three mutations, c.211G > A (p.G71R), c.508_510delTTC (p.F170-) and c.1456 T > G (p.Y486D) in the hotspot regions of the *UGT1A1* gene (exons 1 and 5) in Asian populations, presenting a genotype compatible with clinical picture of CNS-II. The family genetic analysis confirmed the origin of these mutations.

**Conclusion:**

*UGT1A1* gene analysis should be performed in all cases with unexplained unconjugated hyperbilirubinemia. The description of patients with peculiar genotypes especially including family analysis could help explain the relationship between the genotype and phenotype,it is helpful for clinicians to predict the outcome of the patients.

## Background

Crigler-Najjar syndromes (CNS) are autosomal recessive inherited inborn disorders characterized by non-hemolytic unconjugated hyperbilirubinemia. According to serum total bilirubin concentration (STBC), CNS are classified into two types: type I (CNS-I), in which the STBC is more than 25 times that of the normal level ranging from 342 to 684 μmol/L, and type II (CNS-II), in which it is 6-25 times with a range of STBC within 103-342 μmol/L [1, 2].

Genetic variants of UDP-glucuronosyltransferase 1A1 gene (*UGT1A1*) resulting in the absence or decrease of enzyme activity have been reported associating with CNS. Uridine diphosphate glucuronosyltransferase (UGT) isoform 1A1 (*UGT1A1*) enzyme, encoded by *UGT1A1* gene, is the only isoform of UGTs that significantly contributes to the conjugation of bilirubin for the excretion of the bilirubin [3]. In severe CNS-I, the absence of UGT1A1 enzyme activity lead to non-hemolytic unconjugated hyperbilirubinemia, marked jaundice and may cause bilirubin encephalopathy (kernicterus). However, the milder CNS-II patients with an enzyme activity of 10% present milder hyperbilirubinemia, and such patients can survive into adulthood [4].

In this paper we present the case of a Chinese boy with unconjugated hyperbilirubinemia who was diagnosed with CNS-II and a genetic analysis of his family was performed. As a result, three mutations were identified.

## Case presentation

A mainland Chinese 8-month-old boy had a history of intermittent jaundice for more than seven months. He was born at full term from non-consanguineous parents with birth weight of 3700 g. At fifth day of life, jaundice occurred with no known cause and biochemical tests revealed severe unconjugated hyperbilirubinemia with the levels of STBC were 400 μmol/L. For this reason, the child was treated with phototherapy for seven days and jaundice disappeared. However, intermittent onset of jaundice appeared in seven months with the levels of STBC ranging from 45.7 μmol/L to 107.3 μmol/L. Serological tests for hepatocellular integrity, such as ALT (alanine aminotransferase), AST (aspartate aminotransferase), ALP (alkaline phosphatase) activities, serum albumin and total protein concentrations resulted within normal range. An abdominal ultrasound examination was performed without any positive findings. Furthermore, neonatal viral infections, red blood cell (RBC) enzyme abnormality, hematoma, rash, or diseases of his central nervous system were excluded. His mother was in good health while his father presented slight jaundice. His sister, a 3-year-old girl, had a history of neonatal jaundice. After comprehensive consideration of clinical and laboratory findings as well as his family history, we made a diagnosis of CNS-II and deeper laboratory investigations of the *UGT1A1* gene was suggested.

### Analysis of *UGT1A1*mutation

Genomic DNA was isolated from the leucocytes of patients after obtaining informed consent from proband and his families. Primers for polymerase chain reaction (PCR) and direct sequencing of *UGT1A1* gene are shown in Table 1. The sequences of the amplified DNA fragments were determined directly by the use of sequencing primers.

**Table 1:** **Primers used for amplification and sequencing of the coding and promoter’s regions of the** ***UGT1A1*** **gene**

***UGT1A1***region	Forward primer (5′ → 3′)	Reverse primer (5′ → 3′)
Promoter & Exon 1	GAAACCTAATAAAGCTCCACCTTC	TTGCTCAGCATATATCTGGGGC
Exon 2	TCATTTAAAGGGACCACGCC	GGAAAAGCCAAATCTAAGGTTCC
Exon 3 & Exon 4	ACGTAGTGCATACACCCTTG	GAAACAACGCTATTAAATGCTACG
Exon 5	GAAACAGGTTTCCTTTCCCAAG	CAGAGGGGGCACGATACATA

**Figure 1:**
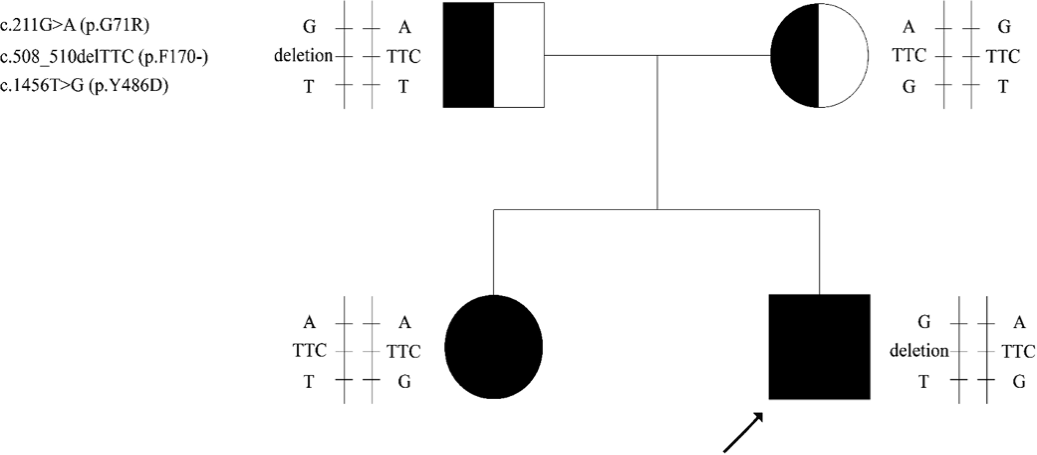
**Pedigree of the Chinese family with three different UGT1A1 mutations.** The proband is indicated by the arrow. The square represents the proband and his father and the circles represent the mother and the sister.

By sequence analysis of entire *UGT1A1* gene, the proband was found to be a genetic compound heterozygote for three mutations. One was a polymorphism with a substitution of G by A at nucleotide position 211 in exon 1 (c.211G > A), resulting in the change of glycine (G) to alanine (R) at codon 71 of the UGT1A1 enzyme (p.G71R). The second one was a 3 bp deletion (TTC) between nucleotides 508 and 510 in exon 1 (c.508_510delTTC), leading to the phenylalanine (F) deletion at position 170 of the UGT1A1 enzyme (p.F170-). The last one was a T to G transition at nucleotide position 1456 in exon 5 (c.1456 T > G), resulting in the substitution of tyrosine (Y) with aspartic acid (D) at codon 486 (p.Y486D). These mutations have been associated with CNS-II except that p.G71R occurred mostly in Gilbert syndrome (a mild form of hereditary *UGT1A1*-associated syndromes). His father was a carrier of heterozygous p.G71R and p.F170-. Heterozygous p.G71R and p.Y486D were detected in his mother. His sister carried homozygous p.G71R and heterozygous p.Y486D. All this hereditary information of families confirmed the inheritance and the allelic distribution of the three mutations (Figure 1). No mutations were detected in any of other exons and screening for the TA duplication in the *UGT1A1* gene promoter of the proband and his family members showed a wild type genotype (6TA/6TA).

## Discussion

UDP-glucuronosyltransferases (UGTs) play a critical role in the detoxification of endogenous and exogenous lipophilic substrates, in particular potentially toxic substrates such as bilirubin, by conjugating them with glucuronic acid and thereby enhancing hydrophilicity for excretion in bile and urine [5]. In humans, UGTs are divided into two families, UGT1 and UGT2, based on amino acid sequence similarity. The UGT1 family comprises nine function enzymes (*UGT1A1*, UGT1A3, UGT1A4, UGT1A5, UGT1A6, UGT1A7, UGT1A8, UGT1A9, and *UGT1A1*0) and four pseudogenes (*UGT1A2P*, *UGT1A11P*, *UGT1A12P*, and *UGT1A13P*), which are encoded by a single gene at 2q37, with each UGT1A isoform consisting of a unique first exon following commonly used exon 2 to 5 [6]. Among all of the UGTs identified up to now, UGT1A1 is the only relevant bilirubin-gulucuronidating enzyme which is mainly expressed in the liver [7]. Genetic variants causing absence, or severe reduction of UGT1A1 activity lead to mild forms of unconjugated hyperbilirubinemia, Gilbert syndrome (GS) and CNS-II, or a severe CNS-I form [8, 9].

In this paper, we found the boy with unconjugated hyperbilirubinemia had a compound heterozygous mutation consisted of three mutations in the *UGT1A1* gene (p.G71R, p.F170- and p.Y486D). Based on the history, along with biochemical and genetics findings, clinical diagnosis of CNS-II was made. According to a previous expression study, UGT1A1activities of the single homozygous model of p.G71R and p.Y486D were 32.2 ± 1.6% and 7.6 ± 0.5% of the wild-type model, respectively. The relative enzyme activity of the double homozygous model of p.G71R and p.Y486D was 6.2 ± 1.6% and that of a heterozygous model of G71R was 60.2 ± 3.5% [10]. Polymorphism p.G71R is the most frequent genetic cause of GS in Asian populations with a frequency of 11%-21% (Single Nucleotide Polymorphism Database [dbSNP]). The missense mutation p.Y486D has been reported previously in CNS-II patients in Japan and China [11, 12]. In previously reported CNS-II cases, the p.Y486D and p.G71R often were observed in the same heterozygous/homozygous state [13–15]. In our report, we found something interesting. The healthy sister of the proband who had a history of neonatal jaundice carried homozygous p.G71R and heterozygous p.Y486D with a suspicion of having GS. However, the mother of the proband in whom heterozygous p.G71R and p.Y486D were identified had always been pretty healthy. This fact leaves us some implications. First, the p.G71R, as a polymorphism, is a mild disease-associated variant without the obvious reduction of the UGT1A1 activity in vivo. Second, the p.Y486D mutation in exon 5, one of the shared exons, is deleterious as it could affect the glucuronidation of all the isoforms (*UGT1A1* to 1A10) expressed from UGT1 family [14]. However, p.Y486D in heterozygous condition needs to combine with some other deleterious mutations to cause clinically CNS-II.

The deletion mutation p.F170-, found in our patient, has been described in CNS-I and CNS-II in Italian population [12, 16]. Deletion of a phenylalanine at codon 170 abolished a conserved double-phenylalanine site in a highly conserved strikingly hydrophobic region between amino acids 161 and 180 (amino-terminal region of the UGT1A1 enzyme). Moreover, exon 1, the location of the deletion mutation, encoded the substrate specific region of *UGT1A1* accounting for 80% of the bilirubin transferase activity. So, this mutation determined CNS-I and CNS-II phenotypes in homozygous and heterozygous conditions, respectively, as the highly deleterious deletion could effectively eliminate the UGT1A1 enzyme activity [16]. In our report, the phenotypes associated with our patient (CNS-II) and his father who all carried heterozygous p.F170- suggested that p.Y486D only in compound heterozygous with deleterious p.F170- could cause obvious jaundice confirmed the recessive inheritance of CNS-II and the compound heterozygote did not determine the complete abolition of UGT1A1 enzyme function, but resulted only in a mild phenotype characterized at variable levels of STBC, with high concentrations at birth followed by a progressive declining over time up to reach values slightly above the normal ranges. And, the clinical phenotype observed was considered compatible with CNS-II. In consideration of the previously reported literatures and our report, we considered that exons 1 and 5 probably were hotspot regions of the *UGT1A1* gene in Asian populations especially in Japanese and Chinese and p.G71R and p.Y486D were two most common variants leading to *UGT1A1* genetically-associated unconjugated hyperbilirubinemia.

## Conclusions

We identified a compound heterozygous mutation, p.G71R/p.F170-/p.Y486D, in *UGT1A1* gene in an 8-month-old Chinese boy with intermittent hyperbilirubinemia who was clinically diagnosed as CNS-II. *UGT1A1* gene analysis should be performed in all cases with unexplained unconjugated hyperbilirubinemia. The description of patients with peculiar genotypes especially including family analysis could help explain the relationship between the genotype and phenotype, helping clinicians predict the outcome of the patients. In addition, as hotspot regions of the *UGT1A1* gene, exons 1 and 5 need to be commonly analyzed in Asian populations especially in Japanese and Chinese.

### Consent

Written informed consent was obtained from the patient’s parents for publication of this Case report and any accompanying images. A copy of the written consent is available for review by the Editor-in-Chief of this journal.

### Ethics

The study protocol was approved by the ethics committee of the Children’s Hospital of Nanjing Medical University.

## Abbreviations

CNS-I: Crigler-Najjar syndrome type I
CNS-II: Crigler-Najjar syndrome type II
GS: Gilbert syndrome
UGTs: UDP-glucuronosyltransferases
STBC: Serum total bilirubin concentration
UGT1A1: UDP-glucuronosyltransferase 1A1.
